# Supplementary material for: ANME-2a perform methane oxidation in brackish coastal sediments via multiple pathways
Source: Sci Adv. 2026 Oct 30;12(44):eaei5868. doi: 10.1126/sciadv.aei5868 (PMC13721985; doi:10.1126/sciadv.aei5868)
Supplement: Supplementary file 1 [file sciadv.aei5868_sm.pdf]

Science Advances
AAAS

Supplementary Materials for

ANME-2a perform methane oxidation in brackish coastal sediments via multiple pathways

Robin Klomp *et al.*

Corresponding author: Caroline P. Slomp, caroline.slomp@ru.nl

Sci. Adv. **12**, eaei5868 (2026)
DOI: 10.1126/sciadv.aei5868

The PDF file includes:

Figs. S1 to S11
Tables S1 to S4
Legend for data file S1
References

Other Supplementary Material for this manuscript includes the following:

Data file S1

Supplementary figures

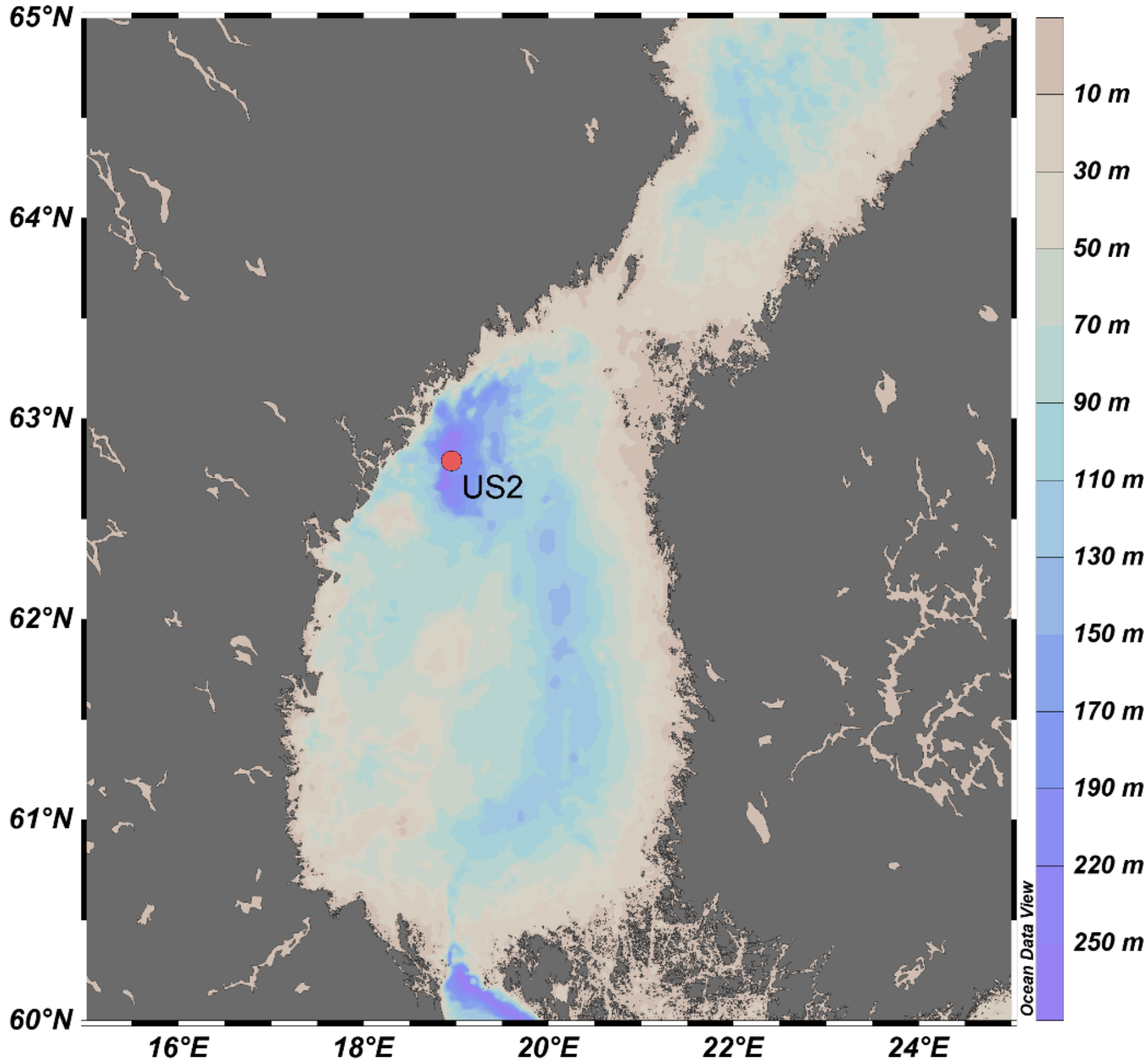

Fig. S1. Map of the Bothnian Sea. The location of site US2 is indicated. The figure was made with Ocean Data View (102).

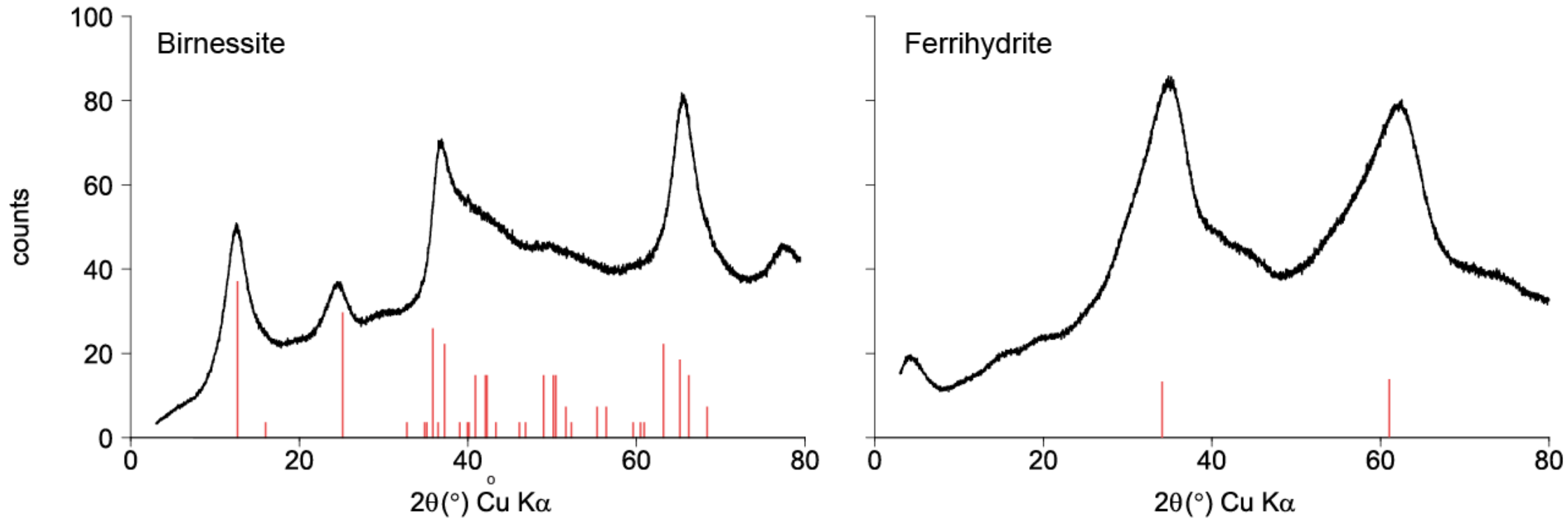

Fig. S2. X-ray diffraction spectra of birnessite and ferrihydrite. The red bars indicate the theoretical positions of the 2θ peaks for birnessite (64) and ferrihydrite (103).

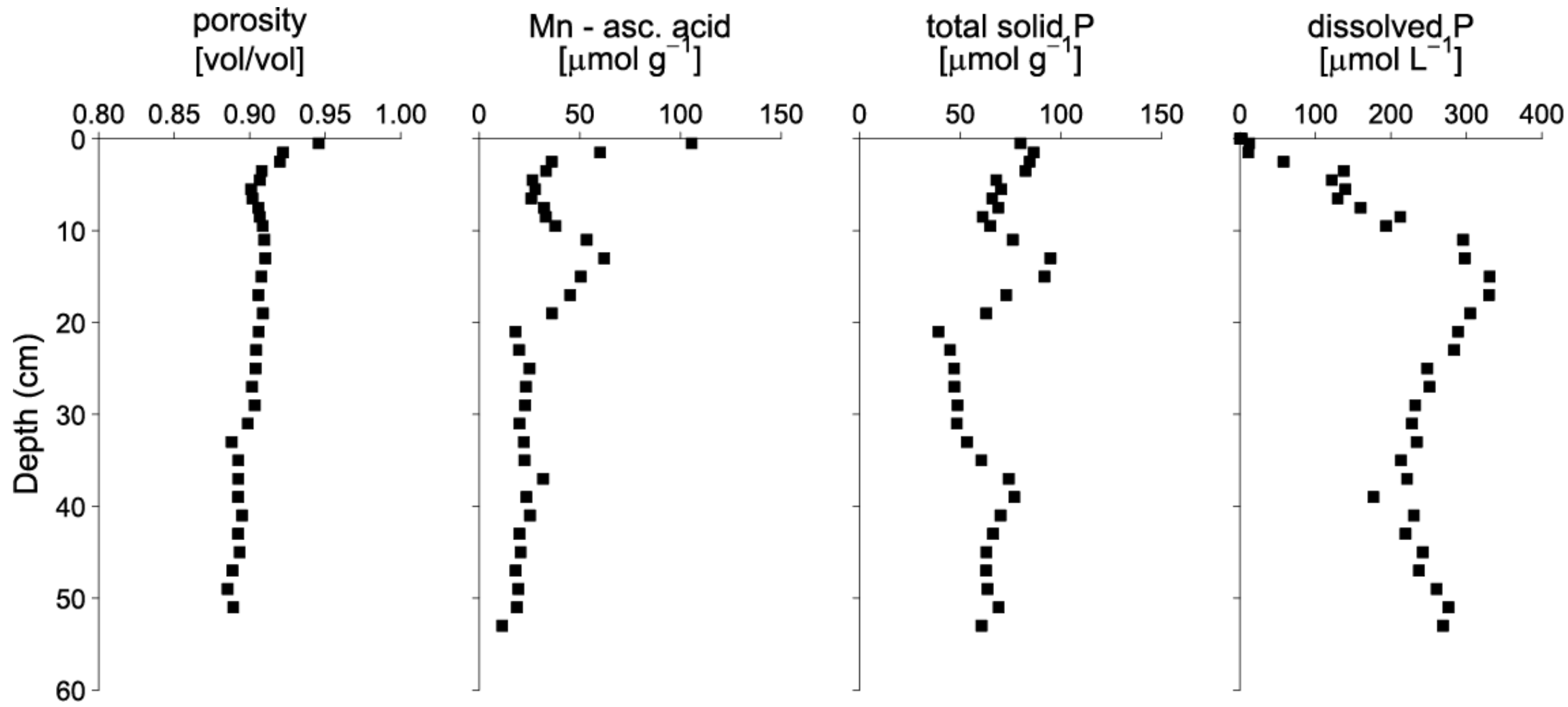

Fig. S3. Sediment profiles of porosity, Mn extracted in ascorbic acid, total solid P and dissolved P. The maximum in Mn extracted in ascorbic acid between depths of 10 and 20 cm corresponds to a maximum in total solid P, which suggests that the peak represents a Mn phosphate phase. This zone also corresponds with the maximum dissolved P concentrations in the pore water.

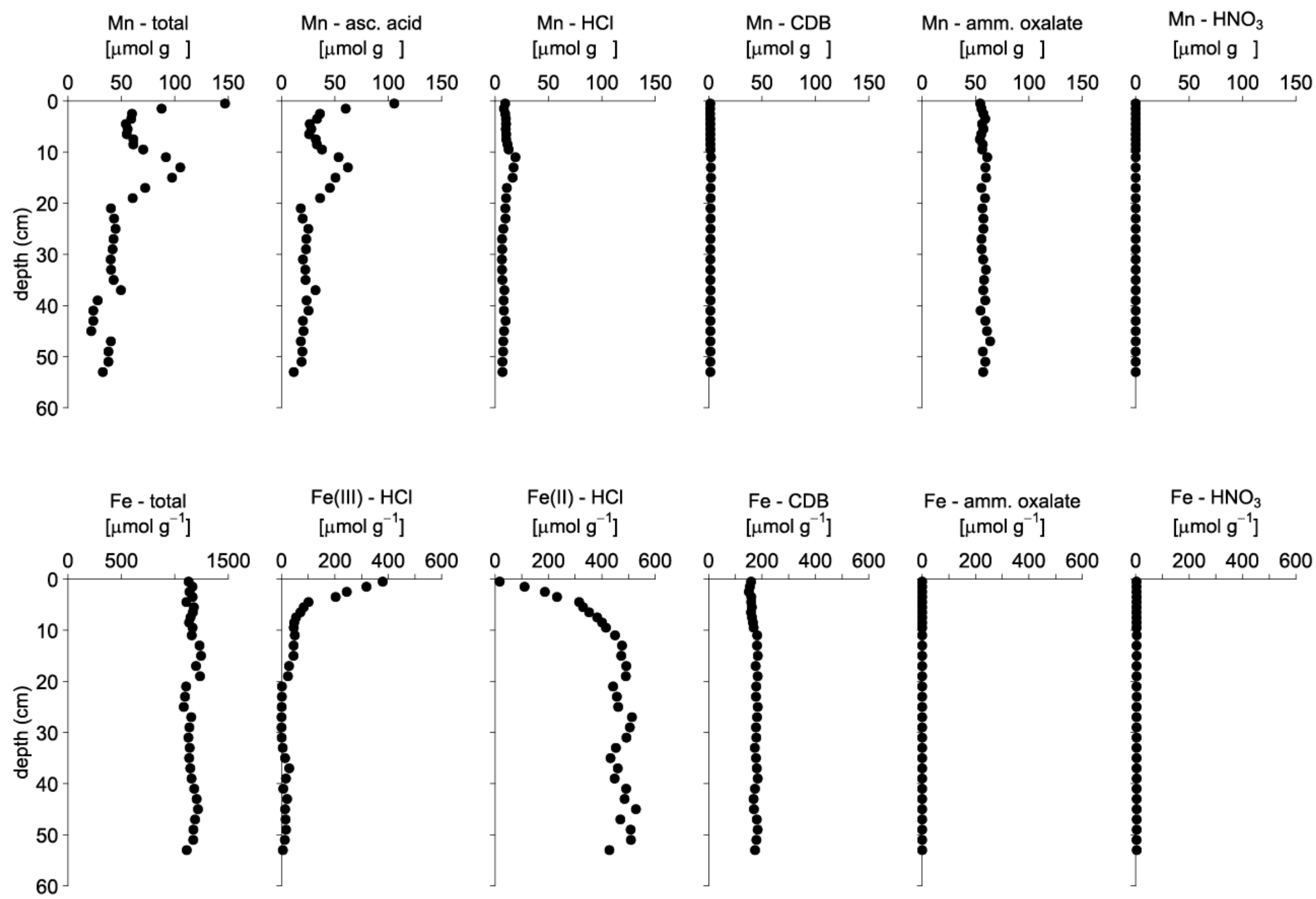

Fig. S4. Sediment profiles of total Mn and Fe and from all steps in the sequential extractions. Ascorbic acid (asc. acid) extracts poorly ordered Mn oxides, HCl extracts Mn carbonates and easily reducible Fe(III) and Fe(II) minerals, sodium dithionite (CDB) extracts crystalline Fe and Mn oxides and Mn bound to clays, ammonium oxalate (amm. oxalate) extracts recalcitrant Fe oxides, crystalline Mn oxides and Mn bound to clays and HNO_3 extracts pyrite, including any associated Mn. Note the different range for the x-axis of Fe total.

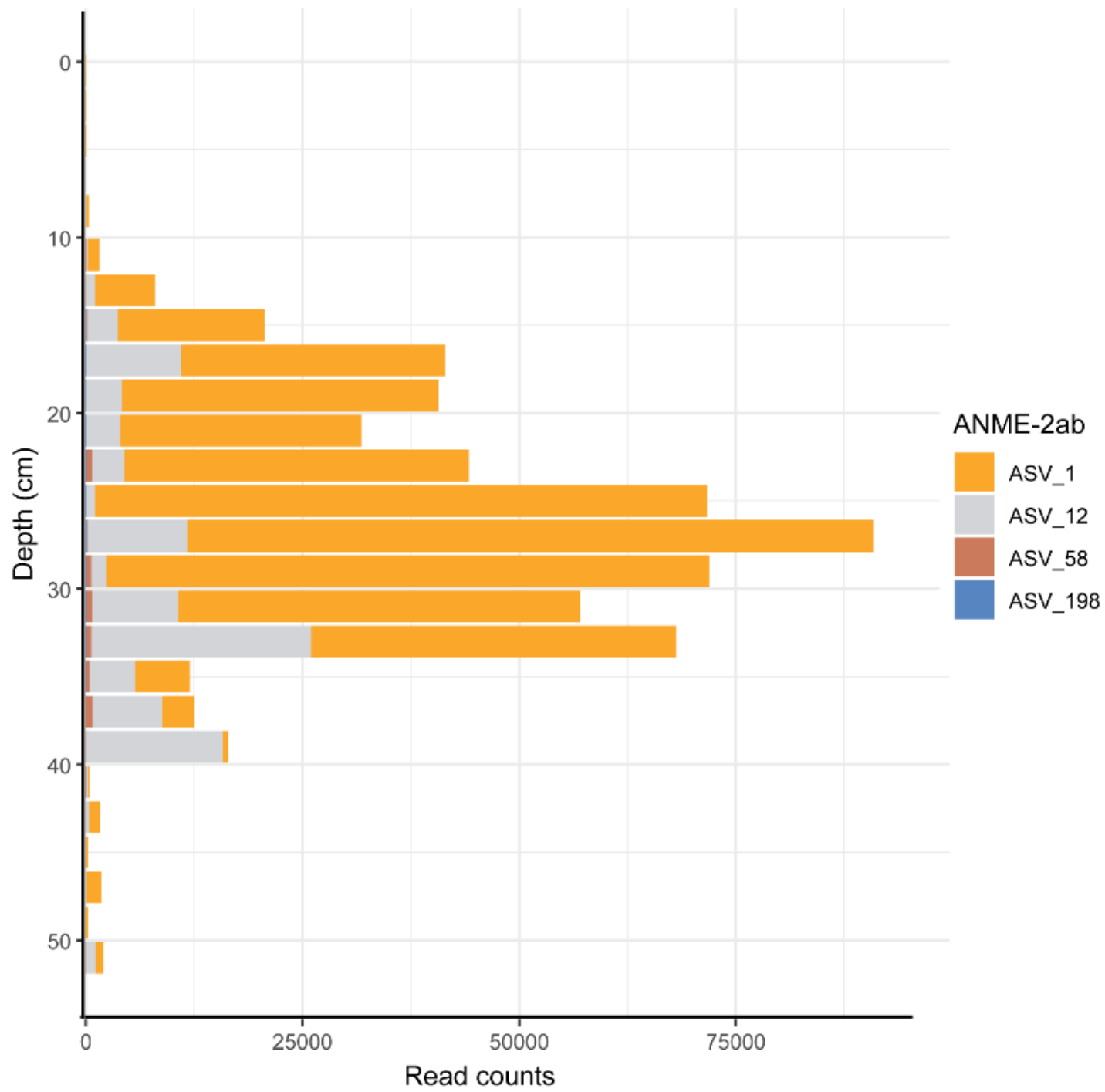

Fig. S5. The total read counts of different ASVs assigned taxonomically to ANME-2ab across the sediment depths.

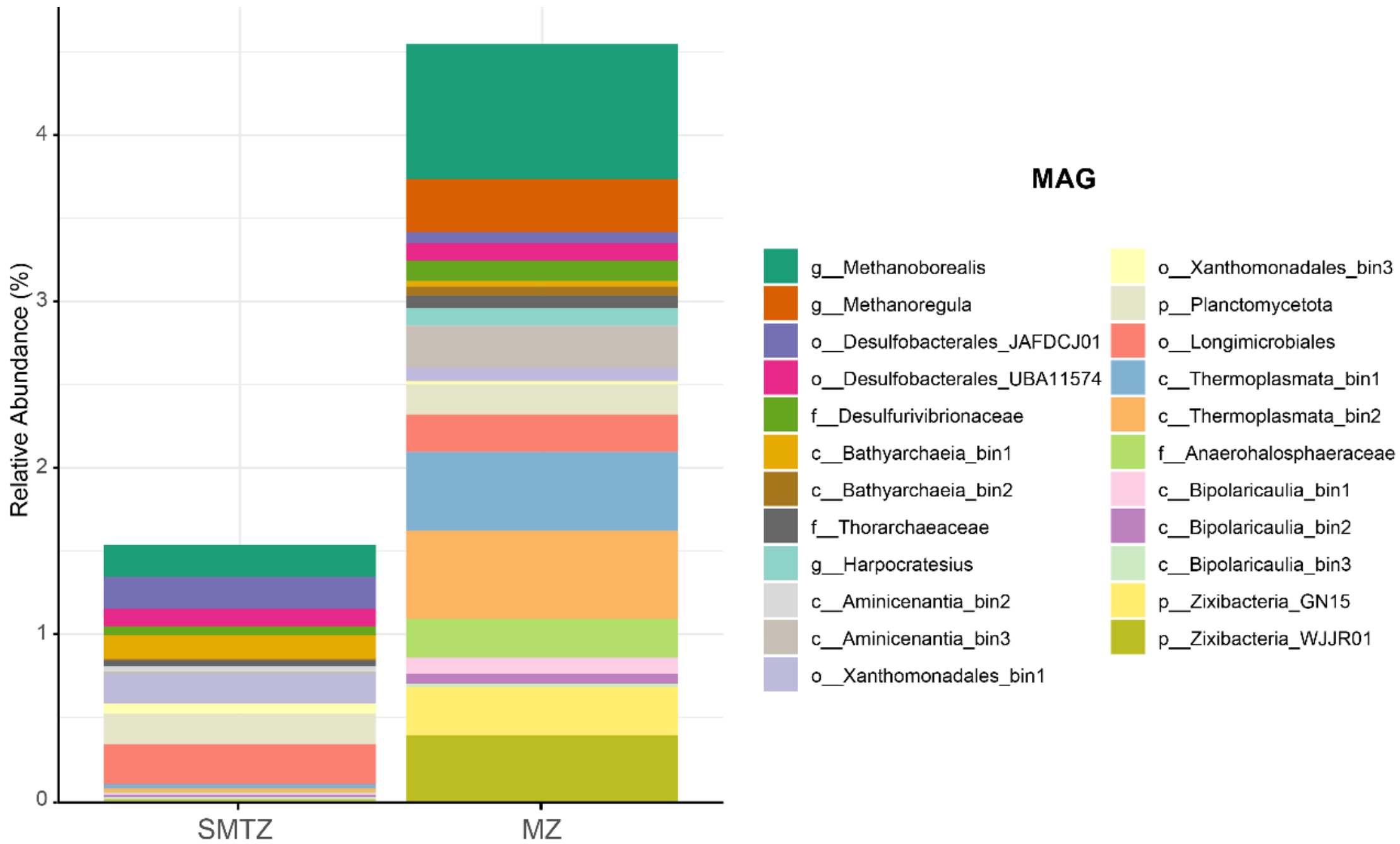

Fig. S6. The MAGs with the highest coverage in metatranscriptome data (Fig. 4) in the SMTZ and below the SMTZ in the methanogenic zone (MZ), portrayed as relative abundance of reads mapped to the metagenome of each depth.

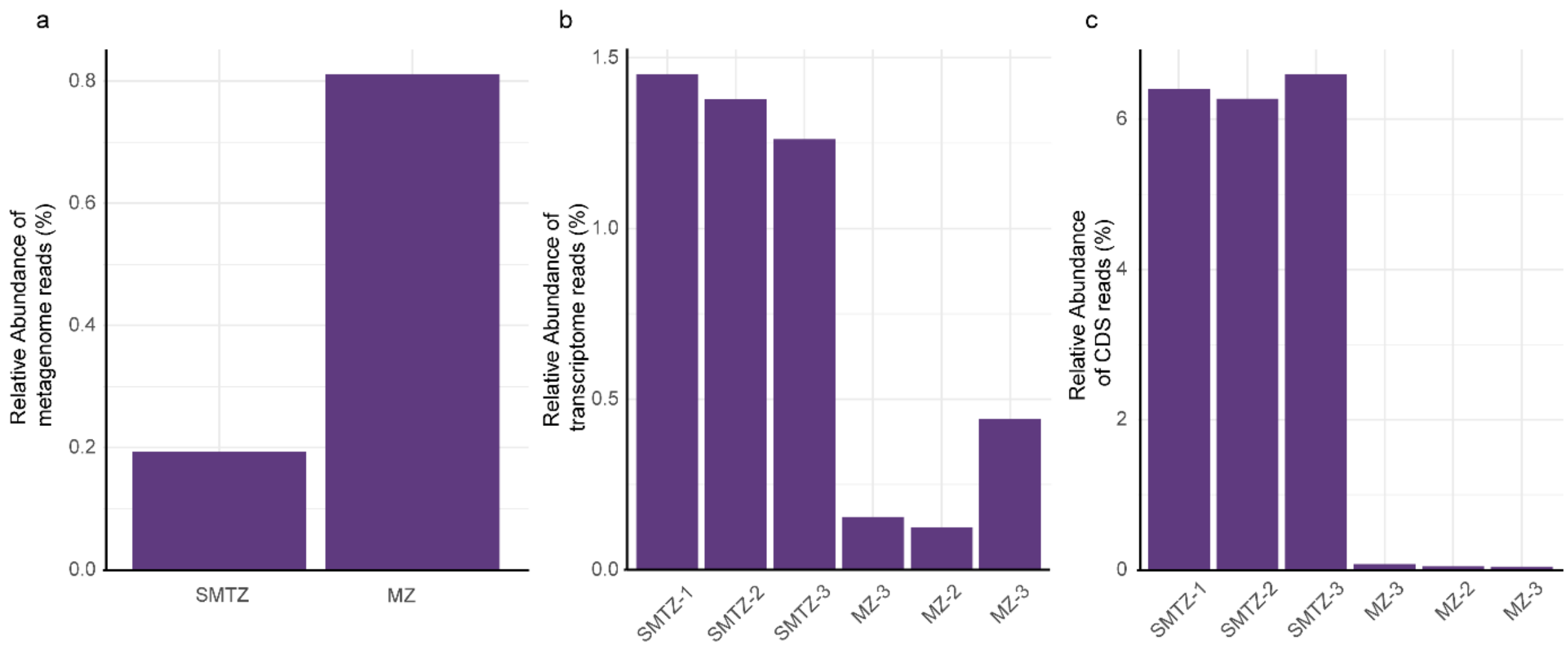

Fig. S7. The abundance of ANME-2a MAG. These were mapped to a) the whole metagenome, b) the whole transcriptome; and c) the relative abundance of ANME-2a MAG CDS reads from all CDS reads mapped to analyzed MAGs in both the SMTZ and below the SMTZ in the methanogenic zone (MZ), portrayed as relative abundance of reads mapped to the metagenome of each depth.

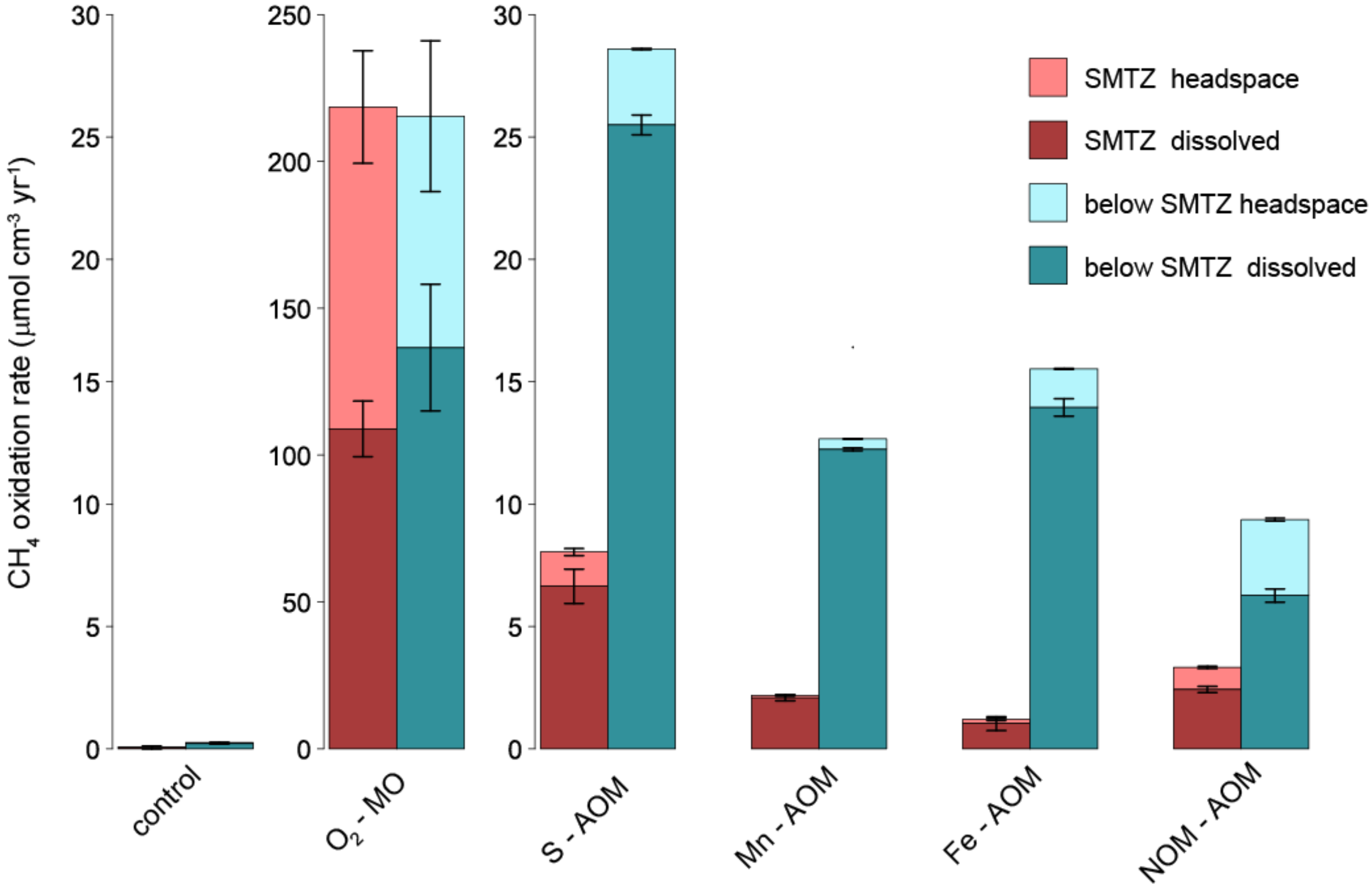

Fig. S8. Maximum methane oxidation rates in the different incubation experiments of sediment from within and below the SMTZ. Rates were calculated based on the increase in ^{13}C-CO_2 and ^{12}C-CO_2 in the headspace (lighter color) and the dissolved phase (darker color).

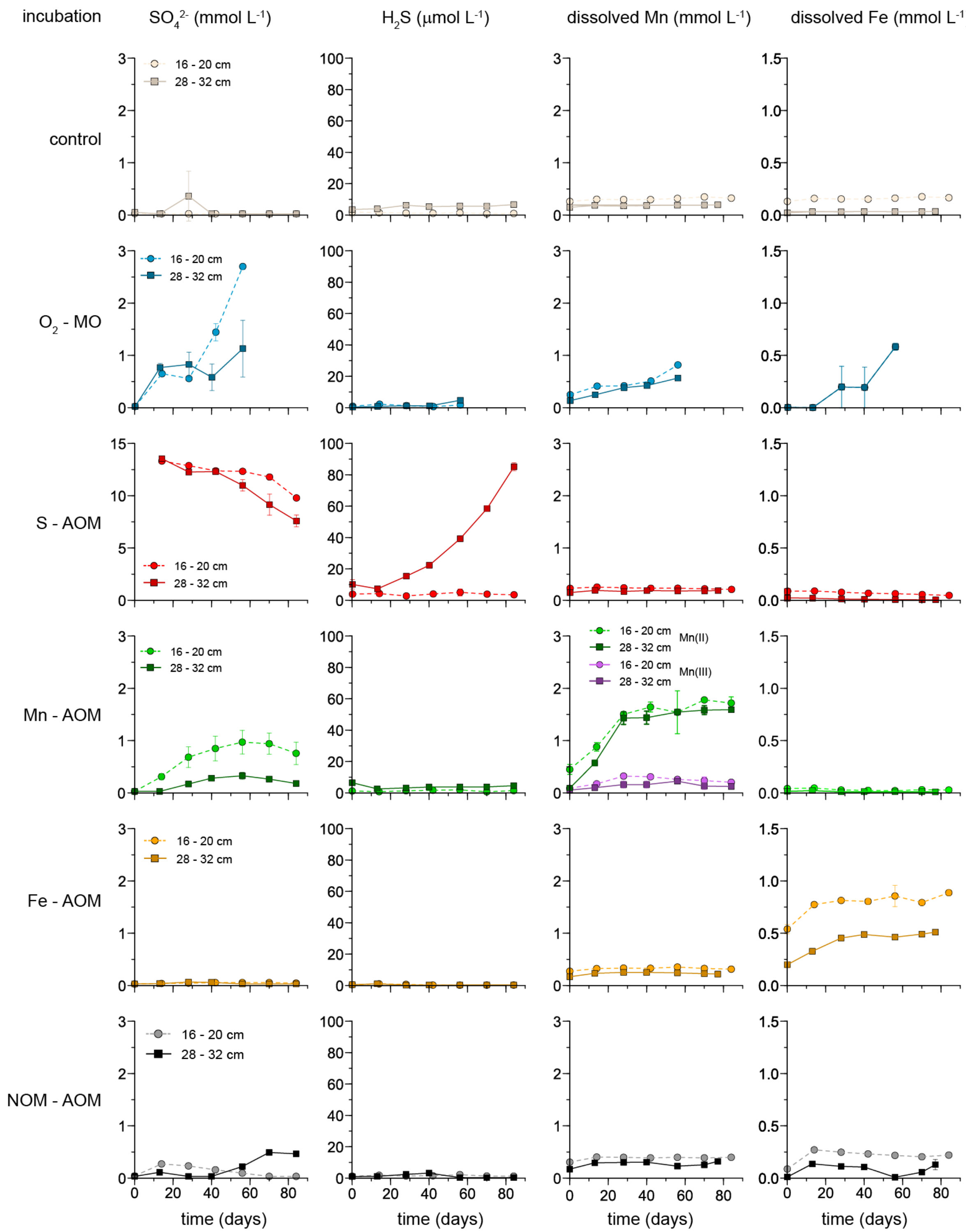

Fig. S9. Concentrations of sulfate, sulfide, dissolved Mn and dissolved Fe in the incubations. Note that, in the Mn-AOM incubation, the redox speciation of dissolved Mn was determined, providing insight into the concentrations of Mn(II) and Mn(III).

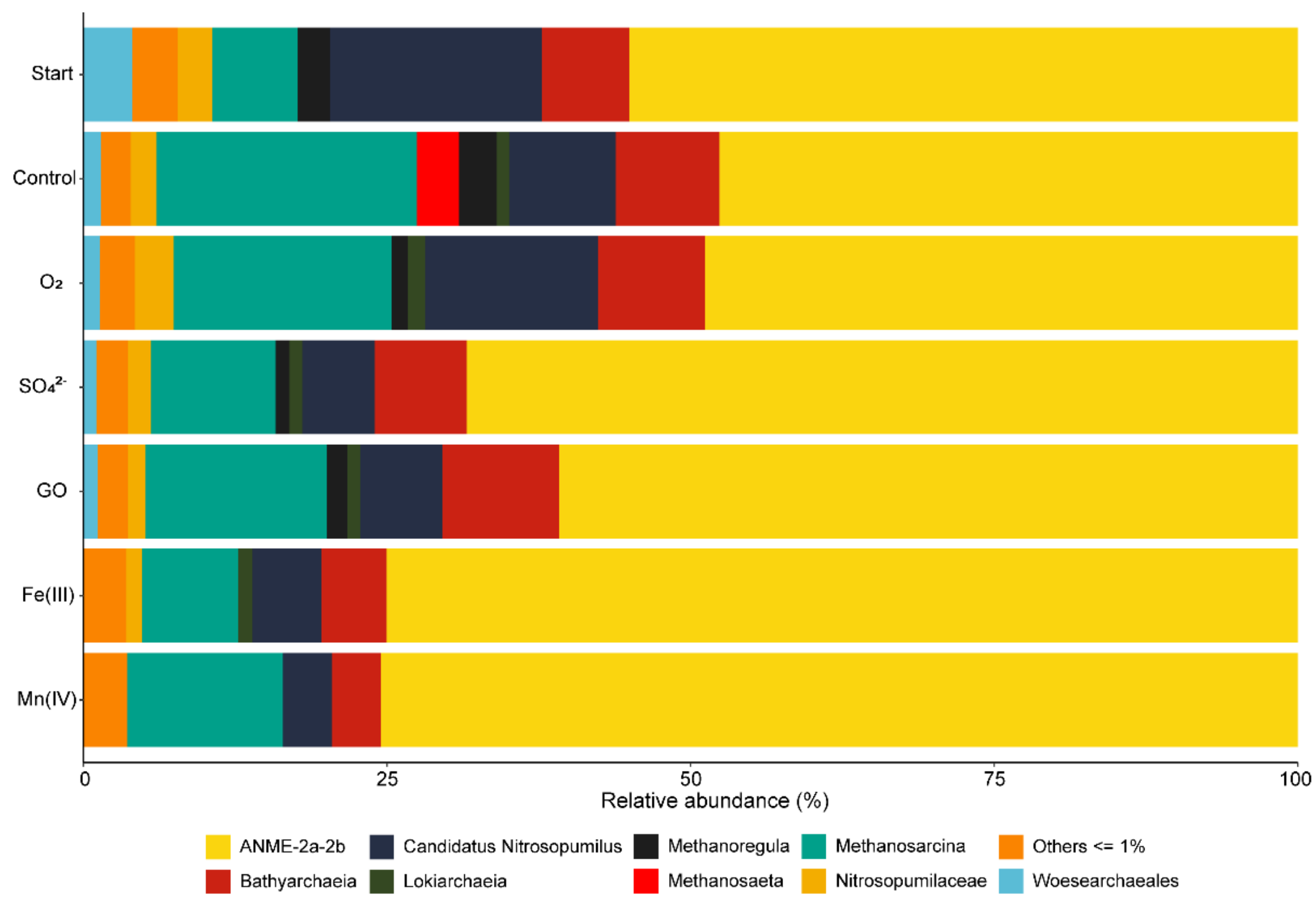

Fig. S10. The relative abundance of archaeal taxa with >1% abundance in all AOM incubations and in the sediment at the start.

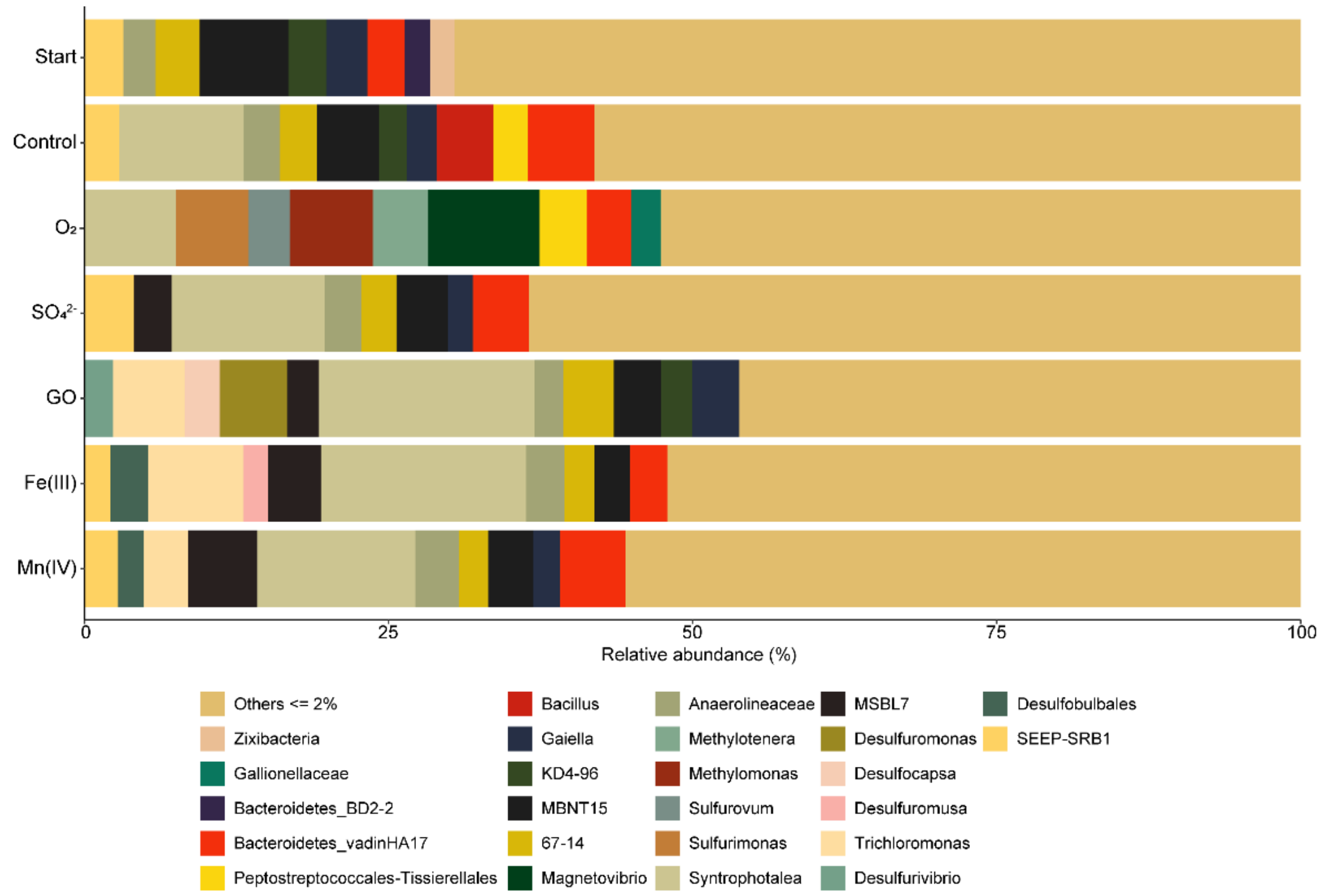

Fig. S11. The relative abundance of bacterial taxa with >1% abundance in all AOM incubations and in the sediment at the start.

Table S1. Scheme for the sediment Mn extraction (64)

Step	Extraction solution	Time of extraction (h)	Mineral phase targeted
1	0.17 M sodium citrate, 0.6 M sodium bicarbonate and 0.057 M ascorbic acid (pH 7.5)	24	Mn oxide and Mn phosphates
2	1 M HCl	4	Mn carbonate and Mn sulfides
3	50 g L^{-1} sodium dithionite solution buffered with 0.35 M acetic acid/0.2 M sodium citrate to pH 48	4	Non-reactive Mn bound to clays
4	0.2 M ammonium oxalate / 0.17 M oxalic acid (pH 3.2)	6	Non-reactive Mn bound to clays
5	65% HNO_3	2	Mn bound to and incorporated in pyrite

Table S2. Scheme for the sediment Fe extraction (65)

Step	Extraction solution	Time of extraction (h)	Mineral phase targeted
1	1 M HCl	4	Poorly ordered or pH-sensitive Fe(III) and Fe(II) minerals (e.g. ferrihydrite, siderite or Fe monosulfide)
2	50 g L^{-1} sodium dithionite solution buffered with 0.35 M acetic acid/0.2 M sodium citrate to pH 48	4	Crystalline Fe oxides (goethite, hematite)
3	0.2 M ammonium oxalate / 0.17 M oxalic acid (pH 3.2)	6	Recalcitrant Fe oxides (magnetite)
4	65% HNO_3	2	Pyrite

Table S3. Composition of the artificial sulfate-free seawater (pH 7.5).

Compound	Concentration
NaCl	5.2 g L^{-1}
$MgCl_2$ * 6 H_2O	1 g L^{-1}
$CaCl_2$ * 2 H_2O	0.28 g L^{-1}
KCl	0.1 g L^{-1}
HEPES	20 mmol L^{-1}

Table S4. Taxonomy and relative abundance of the different ANME MAGs obtained in the metagenomic analysis of the sediment samples, based on SingleM analysis.

Taxonomy	Incubation within SMTZ (16-20 cm)	Incubation below SMTZ (28-32 cm)
Root; d__Archaea; p__Halobacteriota; c__Methanosarcinia; o__Methanosarcinales; f__Methanocomedenaceae; g__Kmv04	0.37	1.71
Root; d__Archaea; p__Halobacteriota; c__Methanosarcinia; o__Methanosarcinales; f__Methanocomedenaceae; g__QBUR01	0	0.01
Root; d__Archaea; p__Halobacteriota; c__Methanosarcinia; o__Methanosarcinales; f__Methanocomedenaceae; g__Methanocomedens	0	0

Supplementary Datafile S1. (separate file)
Dataset corresponding to the figures and tables in the manuscript.

REFERENCES

1. W. S. Reeburgh, Oceanic methane biogeochemistry. *Chem. Rev.* **107**, 486–513 (2007).

2. K. Knittel, A. Boetius, Anaerobic oxidation of methane: Progress with an unknown process. *Annu. Rev. Microbiol.* **63**, 311–334 (2009).

3. L. L. Lapham, K. G. Lloyd, H. Fossing, S. Flury, J. B. Jensen, M. J. Alperin, G. Rehder, W. Holzhueter, T. Ferdelman, B. B. Jørgensen, Methane leakage through the sulfate–methane transition zone of the Baltic seabed. *Nat. Geosci.* **17**, 1277–1283 (2024).

4. O. M. Żygadłowska, F. Roth, N. A. G. M. van Helmond, W. K. Lenstra, J. Venetz, N. Dotsios, T. Röckmann, A. J. Veraart, C. Stranne, C. Humborg, M. S. M. Jetten, C. P. Slomp, Eutrophication and deoxygenation drive high methane emissions from a brackish coastal system. *Environ. Sci. Technol.* **58**, 10582–10590 (2024).

5. L. J. Hamdan, K. P. Wickland, Methane emissions from oceans, coasts, and freshwater habitats: New perspectives and feedbacks on climate *Limnol. Oceanogr.* **61**, S3–S12 (2016).

6. D. J. Burdige, Preservation of organic matter in marine sediments: Controls, mechanisms, and an imbalance in sediment organic carbon budgets. *Chem. Rev.* **107**, 467–485 (2007).

7. P. N. Froelich, G. P. Klinkhammer, M. L. Bender, N. A. Luedtke, G. R. Heath, D. Cullen, P. Dauphin, D. Hammond, B. Hartman, V. Maynard, Early oxidation of organic matter in pelagic sediments of the eastern equatorial Atlantic: Suboxic diagenesis. *Geochim. Cosmochim. Acta* **43**, 1075–1090 (1979).

8. D. E. Canfield, B. Thamdrup, Towards a consistent classification scheme for geochemical environments, or, why we wish the term “suboxic” would go away. *Geobiology* **7**, 385–392 (2009).

9. A. Boetius, K. Ravenschlag, C. J. Schubert, D. Rickert, F. Widdel, A. Gieseke, R. Amann, B. B. Jørgensen, U. Witte, O. Pfannkuche, A marine microbial consortium apparently mediating anaerobic oxidation of methane. *Nature* **407**, 623–626 (2000).

10. V. J. Orphan, C. H. House, K.-U. Hinrichs, K. D. Mckeegan, E. F. Delong, Multiple archaeal groups mediate methane oxidation in anoxic cold seep sediments. *Proc. Natl. Acad. Sci. U.S.A.* **99**, 7663–7668 (2002).

11. A. J. Wallenius, P. Dalcin Martins, C. P. Slomp, M. S. M. Jetten, Anthropogenic and environmental constraints on the microbial methane cycle in coastal sediments. *Front. Microbiol.* **12**, 631621 (2021).

12. E. J. Beal, C. H. House, V. J. Orphan, Manganese- and iron-dependent marine methane oxidation. *Science* **325**, 184–187 (2009).

13. K. E. A. Segarra, C. Comerford, J. Slaughter, S. B. Joye, Impact of electron acceptor availability on the anaerobic oxidation of methane in coastal freshwater and brackish wetland sediments. *Geochim. Cosmochim. Acta* **115**, 15–30 (2013).

14. N. Riedinger, M. J. Formolo, T. W. Lyons, S. Henkel, A. Beck, S. Kasten, An inorganic geochemical argument for coupled anaerobic oxidation of methane and iron reduction in marine sediments. *Geobiology* **12**, 172–181 (2014).

15. M. Egger, O. Rasigraf, C. J. Sapart, T. Jilbert, M. S. M. Jetten, T. Röckmann, C. Van Der Veen, N. Bândă, B. Kartal, K. F. Ettwig, C. P. Slomp, Iron-mediated anaerobic oxidation of methane in brackish coastal sediments. *Environ. Sci. Technol.* **49**, 277–283 (2015).

16. D. A. Aromokeye, A. C. Kulkarni, M. Elvert, G. Wegener, S. Henkel, S. Coffinet, T. Eickhorst, O. E. Oni, T. Richter-Heitmann, A. Schnakenberg, H. Taubner, L. Wunder, X. Yin, Q. Zhu, K.-U. Hinrichs, S. Kasten, M. W. Friedrich, Rates and microbial players of iron-driven anaerobic oxidation of methane in methanic marine sediments. *Front. Microbiol.* **10**, 3041 (2020).

17. W. K. Lenstra, N. A. G. M. van Helmond, P. Dalcin Martins, A. J. Wallenius, M. S. M. Jetten, C. P. Slomp, Gene-based modeling of methane oxidation in coastal sediments: Constraints on the efficiency of the microbial methane filter. *Environ. Sci. Technol.* **57**, 12722–12731 (2023).

18. R. Klomp, A. J. Wallenius, N. A. G. M. van Helmond, W. K. Lenstra, O. M. Żygadłowska, M. S. M. Jetten, C. P. Slomp, Potential for manganese oxide driven anaerobic methane oxidation in sediments of a seasonally euxinic coastal basin. *Estuaries Coasts* **49**, 72 (2026).

19. J. Rooze, M. Egger, I. Tsandev, C. P. Slomp, Iron-dependent anaerobic oxidation of methane in coastal surface sediments: Potential controls and impact. *Limnol. Oceanogr.* **61**, S267–S282 (2016).

20. L. Holmkvist, T. G. Ferdelman, B. B. Jørgensen, A cryptic sulfur cycle driven by iron in the methane zone of marine sediment (Aarhus Bay, Denmark). *Geochim. Cosmochim. Acta* **75**, 3581–3599 (2011).

21. K. Á. Norði, B. Thamdrup, C. J. Schubert, Anaerobic oxidation of methane in an iron-rich Danish freshwater lake sediment. *Limnol. Oceanogr.* **58**, 546–554 (2013).

22. G. Su, J. Zopfi, H. Yao, L. Steinle, H. Niemann, M. F. Lehmann, Manganese/iron-supported sulfate-dependent anaerobic oxidation of methane by archaea in lake sediments. *Limnol. Oceanogr.* **65**, 863–875 (2020).

23. K. F. Ettwig, B. Zhu, D. Speth, J. T. Keltjens, M. S. M. Jetten, B. Kartal, Archaea catalyze iron-dependent anaerobic oxidation of methane. *Proc. Natl. Acad. Sci. U.S.A.* **113**, 12792–12796 (2016).

24. C. Cai, A. O. Leu, G.-J. Xie, J. Guo, Y. Feng, J.-X. Zhao, G. W. Tyson, Z. Yuan, S. Hu, A methanotrophic archaeon couples anaerobic oxidation of methane to Fe(III) reduction. *ISME J.* **12**, 1929–1939 (2018).

25. A. O. Leu, C. Cai, S. J. McIlroy, G. Southam, V. J. Orphan, Z. Yuan, S. Hu, G. W. Tyson, Anaerobic methane oxidation coupled to manganese reduction by members of the Methanoperedenaceae. *ISME J.* **14**, 1030–1041 (2020).

26. O. Rasigraf, N. A. G. M. van Helmond, J. Frank, W. K. Lenstra, M. Egger, C. P. Slomp, M. S. M. Jetten, Microbial community composition and functional potential in Bothnian Sea sediments

is linked to Fe and S dynamics and the quality of organic matter. *Limnol. Oceanogr.* **65**, S113–S133 (2020).

27. A. J. Wallenius, J. Venetz, O. M. Żygadłowska, W. K. Lenstra, N. A. G. M. van Helmond, P. Dalcin Martins, C. P. Slomp, M. S. M. Jetten, A ubiquitous and diverse methanogenic community drives microbial methane cycling in eutrophic coastal sediments. *FEMS Microbiol. Ecol.* **101**, fiaf075 (2025).

28. G. L. Chadwick, C. T. Skennerton, R. Laso-Pérez, A. O. Leu, D. R. Speth, H. Yu, C. Morgan-Lang, R. Hatzenpichler, D. Goudeau, R. Malmstrom, W. J. Brazelton, T. Woyke, S. J. Hallam, G. W. Tyson, G. Wegener, A. Boetius, V. J. Orphan, Comparative genomics reveals electron transfer and syntrophic mechanisms differentiating methanotrophic and methanogenic archaea. *PLOS Biol.* **20**, e3001508 (2022).

29. S. Scheller, H. Yu, G. L. Chadwick, S. E. McGlynn, V. J. Orphan, Artificial electron acceptors decouple archaeal methane oxidation from sulfate reduction. *Science* **351**, 703–4707 (2016).

30. E. I. Valenzuela, A. Prieto-davó, N. E. López-Lozano, A. Hernández-Eligio, L. Vega-Alvarado, K. Juárez, A. S. García-gonzález, M. G. López, F. J. Cervantes, Anaerobic methane oxidation driven by microbial reduction of natural organic matter in a tropical wetland. *Appl. Environ. Microbiol.* **83**, e00645-17 (2017).

31. Y. Zhao, Y. Liu, S. Cao, Q. Hao, C. Liu, Y. Li, Anaerobic oxidation of methane driven by different electron acceptors: A review. *Sci. Total Environ.* **946**, 174287 (2024).

32. Y. N. Bai, X. N. Wang, J. Wu, Y. Z. Lu, L. Fu, F. Zhang, T. C. Lau, R. J. Zeng, Humic substances as electron acceptors for anaerobic oxidation of methane driven by ANME-2d. *Water Res.* **164**, 114935 (2019).

33. S. van Grinsven, J. S. Sinninghe Damsté, L. Villanueva, Assessing the effect of humic substances and Fe(III) as potential electron acceptors for anaerobic methane oxidation in a marine anoxic system. *Microorganisms* **8**, 1288 (2020).

34. K. A. J. Pelsma, N. A. G. M. van Helmond, W. K. Lenstra, T. Röckmann, M. S. M. Jetten, C. P. Slomp, C. U. Welte, Anaerobic methanotrophy is stimulated by graphene oxide in a brackish urban canal sediment. *Environ. Microbiol.* **25**, 3104–3115 (2023).

35. A. J. Wallenius, A. O. Leu, R. Klomp, S. J. McIlroy, G. W. Tyson, C. P. Slomp, M. S. M. Jetten, Potential for metal-coupled methane oxidation by *Candidatus* Methanocomedenaceae in coastal sediments. bioRxiv 712598 [Preprint] (2026). https://doi.org/10.64898/2026.03.20.712598.

36. T. Zhang, X. Xiao, S. Chen, J. Zhao, Z. Chen, J. Feng, Q. Liang, T. J. Phelps, C. Zhang, Active anaerobic archaeal methanotrophs in recently emerged cold seeps of Northern South China Sea. *Front. Microbiol.* **11**, 612135 (2020).

37. M. J. Whiticar, Carbon and hydrogen isotope systematics of bacterial formation and oxidation of methane. *Chem. Geol.* **161**, 291–314 (1999).

38. N. Iversen, B. B. Jorgensen, Anaerobic methane oxidation rates at the sulfate-methane transition in marine sediments from Kattegat and Skagerrak (Denmark). *Limnol. Oceanogr.* **30**, 944–955 (1985).

39. L. Schreiber, T. Holler, K. Knittel, A. Meyerdierks, R. Amann, Identification of the dominant sulfate-reducing bacterial partner of anaerobic methanotrophs of the ANME-2 clade. *Environ. Microbiol.* **12**, 2327–2340 (2010).

40. R. Murali, H. Yu, D. R. Speth, F. Wu, K. S. Metcalfe, A. Crémière, R. Laso-Pèrez, R. R. Malmstrom, D. Goudeau, T. Woyke, R. Hatzenpichler, G. L. Chadwick, S. A. Connon, V. J. Orphan, Physiological potential and evolutionary trajectories of syntrophic sulfate-reducing bacterial partners of anaerobic methanotrophic archaea. *PLOS Biol.* **21**, e3002292 (2023).

41. L. Xu, G. C. Zhuang, A. Montgomery, Q. Liang, S. B. Joye, F. Wang, Methyl-compounds driven benthic carbon cycling in the sulfate-reducing sediments of South China Sea. *Environ. Microbiol.* **23**, 641–651 (2021).

42. A. W. Dale, P. Regnier, N. J. Knab, B. B. Jørgensen, P. Van Cappellen, Anaerobic oxidation of methane (AOM) in marine sediments from the Skagerrak (Denmark): II. Reaction-transport modeling. *Geochim. Cosmochim. Acta* **72**, 2880–2894 (2008).

43. R. Seifert, K. Nauhaus, M. Blumenberg, M. Krüger, W. Michaelis, Methane dynamics in a microbial community of the Black Sea traced by stable carbon isotopes in vitro. *Org. Geochem.* **37**, 1411–1419 (2006).

44. M. Egger, M. Hagens, C. J. Sapart, N. Dijkstra, N. A. G. M. van Helmond, J. M. Mogollón, N. Risgaard-Petersen, C. van der Veen, S. Kasten, N. Riedinger, M. E. Böttcher, T. Röckmann, B. B. Jørgensen, C. P. Slomp, Iron oxide reduction in methane-rich deep Baltic Sea sediments. *Geochim. Cosmochim. Acta* **207**, 256–276 (2017).

45. Y. Gu, M. J. Guberman-Pfeffer, V. Srikanth, C. Shen, F. Giska, K. Gupta, Y. Londer, F. A. Samatey, V. S. Batista, N. S. Malvankar, Structure of Geobacter cytochrome OmcZ identifies mechanism of nanowire assembly and conductivity. *Nat. Microbiol.* **8**, 284–298 (2023).

46. W. K. Lenstra, M. Egger, N. A. G. M. van Helmond, E. Kritzberg, D. J. Conley, C. P. Slomp, Large variations in iron input to an oligotrophic Baltic Sea estuary: Impact on sedimentary phosphorus burial. *Biogeosciences* **15**, 6979–6996 (2018).

47. C. P. Slomp, H. P. Mort, T. Jilbert, D. C. Reed, B. G. Gustafsson, M. Wolthers, Coupled dynamics of iron and phosphorus in sediments of an oligotrophic coastal basin and the impact of anaerobic oxidation of methane. *PLOS ONE* **8**, e62386 (2013).

48. L. Deng, D. Bölsterli, C. Glombitza, B. B. Jørgensen, H. Røy, M. A. Lever, Drivers of methane-cycling archaeal abundances, community structure, and catabolic pathways in continental margin sediments. *Front. Microbiol.* **16**, 1550762 (2025).

49. P. Dalcin Martins, J. P. R. C. de Monlevad, M. J. Echeveste Medrano, W. K. Lenstra, A. J. Wallenius, M. Hermans, C. P. Slomp, C. U. Welte, M. S. M. Jetten, N. A. G. M. van Helmond, Sulfide toxicity as key control on anaerobic oxidation of methane in eutrophic coastal sediments. *Environ. Sci. Technol.* **58**, 11421–11435 (2024).

50. D. Breitburg, L. A. Levin, A. Oschlies, M. Grégoire, F. P. Chavez, D. J. Conley, V. Garçon, D. Gilbert, D. Gutiérrez, K. Isensee, G. S. Jacinto, K. E. Limburg, I. Montes, S. W. A. Naqvi, G. C. Pitcher, N. N. Rabalais, M. R. Roman, K. A. Rose, B. A. Seibel, M. Telszewki, M. Yasuhara, J. Zhang, Declining oxygen in the global ocean and coastal waters. *Science* **359**, eaam7240 (2018).

51. E. A. Canuel, S. S. Cammer, H. A. McIntosh, C. R. Pondell, Climate change impacts on the organic carbon cycle at the land-ocean interface. *Annu. Rev. Earth Planet. Sci.* **40**, 685–711 (2012).

52. X. Xiao, M. Luo, C. Zhang, T. Zhang, X. Yin, X. Wu, J. Zhao, J. Tao, Z. Chen, Q. Liang, X. Dong, Metal-driven anaerobic oxidation of methane as an important methane sink in methanic cold seep sediments. *Microbiol. Spectr.* **11**, e0533722 (2023).

53. K. Nauhaus, M. Albrecht, M. Elvert, A. Boetius, F. Widdel, In vitro cell growth of marine archaeal-bacterial consortia during anaerobic oxidation of methane with sulfate. *Environ. Microbiol.* **9**, 187–196 (2007).

54. A. W. Dale, P. Regnier, P. Van Cappellen, Bioenergetic controls on Anaerobic Oxidation of Methane (AOM) in coastal marine sediments: A theoretical analysis. *Am. J. Sci.* **306**, 246–294 (2006).

55. M. A. Cambon-Bonavita, T. Nadalig, E. Roussel, E. Delage, S. Duperron, J. C. Caprais, A. Boetius, M. Sibuet, Diversity and distribution of methane-oxidizing microbial communities associated with different faunal assemblages in a giant pockmark of the Gabon continental margin. *Deep Sea Res. II Top. Stud. Oceanogr.* **56**, 2248–2258 (2009).

56. S.E. Ruff, H. Kuhfuss, G. Wegener, C. Lott, A. Ramette, J. Wiedling, K. Knittel, M. Weber, Methane seep in shallow-water permeable sediment harbors high diversity of anaerobic methanotrophic communities, Elba, Italy. *Front. Microbiol.* **7**, 374 (2016).

57. R. N. Wallheimer, I. Halevy, O. Sivan, Modeling the controls on microbial iron and manganese reduction in methanic sediments. *Geochim. Cosmochim. Acta* **400**, 32–50 (2025).

58. M. Saunois, A. Martinez, B. Poulter, Z. Zhang, P. A. Raymond, P. Regnier, J. G. Canadell, R. B. Jackson, P. K. Patra, P. Bousquet, P. Ciais, E. J. Dlugokencky, X. Lan, G. H. Allen, D. Bastviken, D. J. Beerling, D. A. Belikov, D. R. Blake, S. Castaldi, M. Crippa, B. R. Deemer, F. Dennison, G. Etiope, N. Gedney, L. Höglund-Isaksson, M. A. Holgerson, P. O. Hopcroft, G. Hugelius, A. Ito, A. K. Jain, R. Janardanan, M. S. Johnson, T. Kleinen, P. B. Krummel, R. Lauerwald, T. Li, X. Liu, Kyle C. Mc Donald, J. R. Melton, J. Mühle, J. Müller, F. Murguia-Flores, Y. Niwa, S. Noce, S. Pan, R. J. Parker, C. Peng, M. Ramonet, W. J. Riley, G. Rocher-Ros, J. A. Rosentreter, M. Sasakawa, A. Segers, S. J. Smith, E. H. Stanley, J. Thanwerdas, H. Tian, A. Tsuruta, F. N. Tubiello, T. S. Weber, Guido R. van der Werf, D. E. J. Worthy, Y. Xi, Y. Yoshida, W. Zhang, B. Zheng, Q. Zhu, Q. Zhu, Q. Zhuang, Global Methane Budget 2000-2020. *Earth Syst. Sci. Data* **17**, 1873–1958 (2025).

59. G. Algesten, L. Brydsten, P. Jonsson, P. Kortelainen, S. Löfgren, L. Rahm, A. Räike, S. Sobek, L. Tranvik, J. Wikner, M. Jansson, Organic carbon budget for the Gulf of Bothnia. *J. Mar. Syst.* **63**, 155–161 (2006).

60. M. Leivuori, L. Niemistö, Sedimentation of trace metals in the Gulf of Bothnia. *Chemosphere* **31**, 3839–3856 (1995).

61. K. Kuliński, G. Rehder, E. Asmala, A. Bartosova, J. Carstensen, B. Gustafsson, P. O. J. Hall, C. Humborg, T. Jilbert, K. Jürgens, H. E. M. Meier, B. Müller-Karulis, M. Naumann, J. E. Olesen, O. Savchuk, A. Schramm, C. P. Slomp, M. Sofiev, A. Sobek, B. Szymczycha, E. Undeman, Biogeochemical functioning of the Baltic Sea. *Earth Syst. Dyn.* **13**, 633–685 (2022).

62. A. B. Josefson, J. Norkko, A. Norkko, Burial and decomposition of plant pigments in surface sediments of the Baltic Sea: Role of oxygen and benthic fauna. *Mar. Ecol. Prog. Ser.* **455**, 33–49 (2012).

63. P. J. M. van Santvoort, G. J. de Lange, J. Thomson, S. Colley, F. J. R. Meysman, C. P. Slomp, Oxidation and origin of organic matter in surficial Eastern Mediterranean hemipelagic sediments. *Aquat. Geochem.* **8**, 153–175 (2002).

64. W. K. Lenstra, R. Klomp, F. Molema, T. Behrends, C. P. Slomp, A sequential extraction procedure for particulate manganese and its application to coastal marine sediments. *Chem. Geol.* **584**, 120538 (2021).

65. P. Kraal, N. Dijkstra, T. Behrends, C. P. Slomp, Phosphorus burial in sediments of the sulfidic deep Black Sea: Key roles for adsorption by calcium carbonate and apatite authigenesis. *Geochim. Cosmochim. Acta* **204**, 140–158 (2017).

66. APHA, *Standard Methods for the Examination of Water and Wastewater* (American Public Health Association, American Water Works Association, Water Environment Federation, 2005).

67. E. D. Burton, L. A. Sullivan, R. T. Bush, S. G. Johnston, A. F. Keene, A simple and inexpensive chromium-reducible sulfur method for acid-sulfate soils. *Appl. Geochem.* **23**, 2759–2766 (2008).

68. M. Brass, T. Röckmann, Continuous-flow isotope ratio mass spectrometry method for carbon and hydrogen isotope measurements on atmospheric methane. *Atmos. Meas. Tech.* **3**, 1707–1721 (2010).

69. C. J. Sapart, C. Van Der Veen, I. Vigano, M. Brass, R. S. W. van de Wal, M. Bock, H. Fischer, T. Sowers, C. Buizert, P. Sperlich, T. Blunier, M. Behrens, J. Schmitt, B. Seth, T. Röckmann, Simultaneous stable isotope analysis of methane and nitrous oxide on ice core samples. *Atmos. Meas. Tech.* **4**, 2607–2618 (2011).

70. J. D. Cline, Spectrophotometric determination of hydrogen sulfide in natural WATERS. *Limnol. Oceanogr.* **14**, 454–458 (1969).

71. L. Solórzano, Determination of ammonia in natural waters by the phenolhypochlorite method 1 1 This research was fully supported by U.S. Atomic Energy Commission Contract No. ATS (11-1) GEN 10, P.A. 20. *Limnol. Oceanogr.* **14**, 799–801 (1969).

72. H. Fossing, B. B. Jørgensen, Measurement of bacterial sulfate reduction in sediments: Evaluation of a single-step chromium reduction method. *Biogeochemistry* **8**, 205–222 (1989).

73. J. Kallmeyer, T. G. Ferdelman, A. Weber, H. Fossing, B. B. Jørgensen, A cold chromium distillation procedure for radiolabeled sulfide applied to sulfate reduction measurements. *Limnol. Oceanogr. Methods* **2**, 171–180 (2004).

74. M. Egger, W. K. Lenstra, D. Jong, F. J. R. Meysman, C. J. Sapart, C. Van Der Veen, T. Röckmann, S. Gonzalez, C. P. Slomp, Rapid sediment accumulation results in high methane effluxes from coastal sediments. *PLOS ONE* **11**, e0161609 (2016).

75. B. B. Jørgensen, T. Fenchel, The sulfur cycle of a marine sediment model system. *Mar. Biol.* **24**, 189–201 (1974).

76. K. Takai, K. Horikoshi, Rapid detection and quantification of members of the archaeal community by quantitative PCR using fluorogenic probes. *Appl. Environ. Microbiol.* **66**, 5066–5072 (2000).

77. D. P. R. Herlemann, M. Labrenz, K. Jürgens, S. Bertilsson, J. J. Waniek, A. F. Andersson, Transitions in bacterial communities along the 2000 km salinity gradient of the Baltic Sea. *ISME J.* **5**, 1571–1579 (2011).

78. J. G. Caporaso, C. L. Lauber, W. A. Walters, D. Berg-Lyons, J. Huntley, N. Fierer, S. M. Owens, J. Betley, L. Fraser, M. Bauer, N. Gormley, J.A. Gilbert, G. Smith, R. Knight, Ultra-high-throughput microbial community analysis on the Illumina HiSeq and MiSeq platforms, *ISME J.* **6**, 1621–1624 (2012).

79. M. M. Weinstein, A. Prem, M. Jin, S. Tang, J. M. Bhasin, FIGARO: An efficient and objective tool for optimizing microbiome rRNA gene trimming parameters. bioRxiv 610394 [Preprint] (2019). https://doi.org/10.1101/610394.

80. B. J. Callahan, P. J. McMurdie, M. J. Rosen, A. W. Han, A. J. A. Johnson, S. P. Holmes, DADA2: High-resolution sample inference from Illumina amplicon data. *Nat. Methods* **13**, 581–583 (2016).

81. S. Andrews, FastQC (2010); www.bioinformatics.babraham.ac.uk/projects/fastqc/.

82. B. J. Woodcroft, S. T. N. Aroney, R. Zhao, M. Cunningham, J. A. M. Mitchell, L. Blackall, G. W. Tyson, SingleM and Sandpiper: Robust microbial taxonomic profiles from metagenomic data. bioRxiv 578060 [Preprint] (2024). https://doi.org/10.1101/2024.01.30.578060.

83. R.J.P. Newell, S.T.N. Aroney, J. Zaugg, P. Sternes, G.W. Tyson, B.J. Woodcroft, "Aviary: Hybrid assembly and genome recovery from metagenomes with Aviary (v0.11.0)," Zenodo (2025); https://zenodo.org/records/14722147.

84. S. Chen, Y. Zhou, Y. Chen, J. Gu, Fastp: An ultra-fast all-in-one FASTQ preprocessor. *Bioinformatics* **34**, i884–i890 (2018).

85. S. Nurk, D. Meleshko, A. Korobeynikov, P. A. Pevzner, MetaSPAdes: A new versatile metagenomic assembler. *Genome Res.* **27**, 824–834 (2017).

86. S.T.N. Aroney, R.J.P. Newell, J.N. Nissen, A.P. Camargo, G.W. Tyson, B.J. Woodcroft, CoverM: Read alignment statistics for metagenomics. *Bioinformatics* **41**, btaf147 (2025).

87. D. D. Kang, J. Froula, R. Egan, Z. Wang, MetaBAT, an efficient tool for accurately reconstructing single genomes from complex microbial communities. *PeerJ* **3**, e1165 (2015).

88. D. D. Kang, F. Li, E. Kirton, A. Thomas, R. Egan, H. An, Z. Wang, MetaBAT 2: An adaptive binning algorithm for robust and efficient genome reconstruction from metagenome assemblies. *PeerJ* **7**, e7359 (2019).

89. S. Pan, C. Zhu, X. M. Zhao, L. P. Coelho, A deep siamese neural network improves metagenome-assembled genomes in microbiome datasets across different environments. *Nat. Commun.* **13**, 2326 (2022).

90. C. M. K. Sieber, A. J. Probst, A. Sharrar, B. C. Thomas, M. Hess, S. G. Tringe, J. F. Banfield, Recovery of genomes from metagenomes via a dereplication, aggregation and scoring strategy. *Nat. Microbiol.* **3**, 836–843 (2018).

91. P. A. Chaumeil, A. J. Mussig, P. Hugenholtz, D. H. Parks, GTDB-Tk v2: Memory friendly classification with the genome taxonomy database. *Bioinformatics* **38**, 5315–5316 (2022).

92. M. Albertsen, P. Hugenholtz, A. Skarshewski, K. L. Nielsen, G. W. Tyson, P. H. Nielsen, Genome sequences of rare, uncultured bacteria obtained by differential coverage binning of multiple metagenomes. *Nat. Biotechnol.* **31**, 533–538 (2013).

93. M. Shaffer, M. A. Borton, B. B. McGivern, A. A. Zayed, S. L. La Rosa, L. M. Solden, P. Liu, A. B. Narrowe, J. Rodríguez-Ramos, B. Bolduc, M. C. Gazitúa, R. A. Daly, G. J. Smith, D. R. Vik, P. B. Pope, M. B. Sullivan, S. Roux, K. C. Wrighton, DRAM for distilling microbial metabolism to automate the curation of microbiome function. *Nucleic Acids Res.* **48**, 8883–8900 (2020).

94. G. Cremers, M. S. M. Jetten, H. J. M. Op den Camp, S. Lücker, Metascan: METabolic Analysis, SCreening and ANnotation of Metagenomes. *Front. Bioinform.* **2**, 861505 (2022).

95. A.I. Garber, K.H. Nealson, A. Okamoto, S.M. McAllister, C.S. Chan, R.A. Barco, N. Merino, FeGenie: A comprehensive tool for the identification of iron genes and iron gene neighborhoods in genome and metagenome assemblies. *Front. Microbiol.* **11**, 37 (2020).

96. D. Hyatt, G.-L. Chen, P. F. Locascio, M. L. Land, F. W. Larimer, L. J. Hauser, Prodigal: Prokaryotic gene recognition and translation initiation site identification. *BMC Bioinformatics* **11**, 119 (2010).

97. M. Händel, T. Rennert, K. U. Totsche, A simple method to synthesize birnessite at ambient pressure and temperature. *Geoderma* **193**, 117–121 (2013).

98. K. P. Raven, A. Jain, R. H. Loeppert, Arsenite and arsenate adsorption on ferrihydrite: Kinetics, equilibrium, and adsorption envelopes. *Environ. Sci. Technol.* **32**, 344–349 (1998).

99. B. Silburn, S. Kröger, E. R. Parker, D. B. Sivyer, N. Hicks, C. F. Powell, M. Johnson, N. Greenwood, Benthic pH gradients across a range of shelf sea sediment types linked to sediment characteristics and seasonal variability. *Biogeochemistry* **135**, 69–88 (2017).

100. A. S. Madison, B. M. Tebo, G. W. Luther III, Simultaneous determination of soluble manganese(III), manganese(II) and total manganese in natural (pore)waters. *Talanta* **84**, 374–381 (2011).

101. V. E. Oldham, S. M. Owings, M. R. Jones, B. M. Tebo, G. W. Luther III, Evidence for the presence of strong Mn(III)-binding ligands in the water column of the Chesapeake Bay. *Mar. Chem.* **171**, 58–66 (2015).

102. R. Schlitzer, Ocean Data View (2023).

103. S. Das, M. J. Hendry, J. Essilfie-Dughan, Transformation of two-line ferrihydrite to goethite and hematite as a function of pH and temperature. *Environ. Sci. Technol.* **45**, 268–275 (2011).
